# Omitting SI from depression research: an ethical analysis of PHQ-8 and PHQ-9

**DOI:** 10.1192/j.eurpsy.2026.11293

**Published:** 2026-07-31

**Authors:** E. van Reekum, R. Van Lieshout, L. Schwartz

**Affiliations:** 1 McMaster University; 2 Population Health Research Institute, Hamilton, Canada

## Abstract

**Introduction:**

Validated depression scales are ubiquitous in research. Some include a suicidal ideation (SI) question while others omit it. Safety protocols are often mandated when assessing SI in research, but little guidance exists on the implications of choosing scales that omit SI. Researchers and Ethics Boards therefore decide on a case-by-case basis whether studies using these scales are ethically sound, leading to delays, inconsistent practices, variable care, and accountability concerns if a participant dies by suicide.

**Objectives:**

To explore whether it is ethically justifiable to choose depression scales that omit SI for research, using the PHQ-8 (omits SI) and PHQ-9 as examples.

**Methods:**

We reviewed the scales’ origins and analysed the 100 most recent studies using either PHQ-8 or PHQ-9, assessing design, outcomes, populations, rationales, and safety protocols. We then synthesized arguments for each scale using principles of concern for welfare, respect for persons, and justice, supported by ethics and suicide literature.

**Results:**

PHQ-8 was derived from PHQ-9 for contexts where suicide risk was negligible, depression was a secondary outcome, or data were self-reported.

We found that PHQ-8 is widely used, including as a primary outcome in trials and in higher-risk populations. Study-reported rationales for PHQ-8 use included maintaining anonymity, difficulty providing clinical follow-up, or strong correlation with PHQ-9. 12% of studies reported safety protocols.

Ethical arguments for PHQ-8 similarly focus on protecting confidentiality, minimizing distress from unneeded referrals and unmet care expectations, especially in low-resourced settings or where legal barriers exist, and enabling research when safety protocols are less feasible.

In contrast, ethical arguments for PHQ-9 centre on the additional value of SI data in capturing all core depression symptoms, improving transparency, validity and generalizability. It improves detection of suffering without increasing suicide risk and prioritizes safety, strengthening public trust in research.

**Conclusions:**

The PHQ-8 was designed and is used primarily to improve study feasibility and avoid ethical and logistical complexities associated with SI disclosure, such as perceived accountability or obligation to intervene. While understandable motivations, they risk a false sense of reassurance – avoiding SI questions has not been shown to reduce suicide risk, nor does it preclude the ethical imperative to safeguard participants in studies examining depression. We recommend using scales that include SI, paired with tailored and feasible safety protocols and communication during informed consent about limits of confidentiality and the option to skip questions. If a scale without SI is chosen, its use should be grounded in participant-centered ethics (eg risk of harm from SI disclosure in specific contexts), rather than psychometric equivalence or to circumvent safety protocols.

**Disclosure of Interest:**

None Declared

